# Impact of mesenteric defect closure technique on complications after gastric bypass

**DOI:** 10.1007/s00423-018-1684-z

**Published:** 2018-06-01

**Authors:** Erik Stenberg, Ingmar Näslund, Eva Szabo, Johan Ottosson

**Affiliations:** Department of Surgery, Faculty of Medicine and Health, Örebro University, SE-70185 Örebro, Sweden

**Keywords:** Bariatric surgery, Postoperative complication, Small bowel obstruction, Internal hernia

## Abstract

**Background:**

Closure of mesenteric defects during laparoscopic gastric bypass surgery markedly reduces the risk for small bowel obstruction due to internal hernia. However, this procedure is associated with an increased risk for early small bowel obstruction and pulmonary complication. The purpose of the present study was to evaluate whether the learning curve and subsequent adaptions made to the technique have had an effect on the risk for complications.

**Methods:**

The results of patients operated with a primary laparoscopic gastric bypass procedure, including closure of the mesenteric defects with sutures, during a period soon after introduction (January 1, 2010–December 31, 2011) were compared to those of patients operated recently (January 1, 2014–June 30, 2017). Data were retrieved from the Scandinavian Obesity Surgery Registry (SOReg). The main outcome was reoperation for small bowel obstruction within 30 days after surgery.

**Results:**

A total of 5444 patients were included in the first group (period 1), and 1908 in the second group (period 2). Thirty-day follow-up rates were 97.1 and 97.5% respectively. The risk for early (within 30 days) small bowel obstruction was lower in period 2 than in period 1 (13/1860, 0.7% vs. 67/5285, 1.3%, OR 0.55 (0.30–0.99), *p* = 0.045). The risk for pulmonary complication was also reduced (5/1860, 0.3%, vs. 41/5285, 0.8%, OR 0.34 (0.14–0.87), *p* = 0.019).

**Conclusion:**

Closure of mesenteric defects during laparoscopic gastric bypass surgery can be performed safely and should be viewed as a routine part of that operation.

## Introduction

Gastric bypass is a well-accepted bariatric surgical method to markedly reduce the long-term effects of morbid obesity on cardiovascular disease, cancer development, diabetes, and quality-of-life [1–4]. The development of a laparoscopic technique for gastric bypass has improved recovery and reduced severe postoperative complication and mortality rates [5, 6]. With the introduction of laparoscopic gastric bypass surgery, the number of patients suffering from internal hernia with small bowel obstruction increased dramatically [7]. Presentation, symptoms, and clinical diagnosis differ from those of postoperative bowel obstruction in other groups of patients [8–10], and delay in diagnosis and treatment may result in devastating consequences [10–12]. The risk, however, is markedly reduced if mesenteric defects are closed during the laparoscopic gastric bypass procedure [13]. However, mesenteric defect closure is associated with an increased risk for early small bowel obstruction due to kinking of the jejunojejunostomy [13]. One component of this risk scenario may be a result of being on the early part of the learning curve. Should this be the case, then this is perhaps the price we must pay to reduce the long-term risk for internal hernia formation. Since the general introduction of mesenteric defect closure to bariatric surgical practice in Sweden, some adaptions have been made to the procedure in order to reduce the risk for jejunojejunostomy kinking. The purpose of this study was to see if the complication risk related to mesenteric defect closure has decreased with time, and to describe possible measures that may be taken in order to further reduce the risk.

## Methods

Data were collected from the Scandinavian Obesity Surgery Registry (SOReg), a national quality and research registry for bariatric surgery covering basically all bariatric surgical procedures in Sweden [14, 15]. All patients operated with a primary laparoscopic gastric bypass procedure between January 1, 2010 and June 30, 2017 were eligible for inclusion in the study. Retrocolic gastric bypass procedures, non-closure of the mesenteric defects, closure with methods other than sutures, or method unknown was excluded from the study. Two study groups were constructed, one representing the early period shortly after introduction of the mesenteric defect closure technique (January, 1 2010–December 31, 2011: period 1) and one representing the current situation after establishment of the technique and with adaptions made to the procedure (January 1, 2014–June 30, 2017: period 2). Patients operated between these time periods were excluded from the study.

### Surgical technique

The surgical technique for laparoscopic gastric bypass surgery is highly standardized in Sweden, with 99% being operated with the antecolic-antegastric, so-called Lönroth technique [15, 16]. The technique used for mesenteric defect closure is not so well standardized, but when sutures are used, the mesenteric defects beneath the jejunojejunostomy and at Petersen’s space are predominantly closed using running, non-absorbable sutures [13]. The following two links illustrate the technique for closure of the mesenteric defects using non-absorbable sutures. This is the link to the video demonstrating closure of the mesenteric defect beneath the jejunojejunostomy: https://s3m.io/yZTGe. This is the link to the video demonstrating closure of Petersen Space: https://s3m.io/RcFQy

### Definitions

Comorbidity was defined as a condition requiring continuous medical treatment or continuous positive airway pressure treatment, and specified as sleep apnea, hypertension, diabetes, dyslipidemia, depression, dyspepsia/GERD, or other (in this case specified) condition. History of smoking and previous venous thromboembolism was registered from May 1, 2010.

### Outcomes

The main outcome was reoperation for small bowel obstruction occurring within 30 days after surgery. Secondary endpoints were the occurrence of any intraoperative adverse event, any postoperative complication, serious postoperative complication, or specified postoperative complication other than small bowel obstruction. Specific complications were anastomotic leakage or intraabdominal abscess, bleeding, deep intra-abdominal infection or abscess, gastrointestinal obstruction or ileus, anastomotic stricture, marginal ulcer, port-related complication, cardiovascular event, pulmonary complication (other than pulmonary embolism), venous thromboembolism, urinary tract infection, and other (in this case specified) complication.

All postoperative complications were graded according to the Clavien-Dindo scale [17], with any deviance from a normal postoperative course considered a postoperative complication. A complication graded as Clavien-Dindo Grade 3b or more (i.e., a complication requiring intervention under general anesthesia, or resulting in organ failure or death of the patient) was considered a serious postoperative complication.

### Statistical analyses

The chi-square test was used to evaluate statistical significance for categorical variables. Continuous variables were analyzed using the Student *t* test. Logistic regression was used to evaluate risk for postoperative complication, with odds ratios (OR) and 95% confidence intervals (95% CI) as measures of association. Odds ratios were analyzed unstandardized and standardized for body mass index, age, and sex. A *p* value < 0.05 was considered to be statistically significant.

### Ethical considerations

The study was conducted in accordance with the standards of the 1964 Helsinki Declaration and its later amendments and was approved by the Regional Ethics Committee in Uppsala.

## Results

From January 1, 2010 until December 31, 2011, 5444 primary laparoscopic gastric bypass procedures with closure of the mesenteric defects using running, non-absorbable sutures were identified. These patients were included in the introduction period group (period 1). From January 1, 2014 until June 30, 2017, 1908 primary laparoscopic gastric bypass procedures with closure of the mesenteric defects using non-absorbable sutures were identified. These patients were included in the established technique group (period 2). Follow-up at 30 days after surgery was registered in the SOReg for 5285 patients during period 1 (97.1%) and 1860 during period 2 (97.5%).

### Baseline characteristics

Patients operated during period 1 had a higher BMI and more often comorbid disease than patients operated during period 2 (Table 1). Preoperative weight reduction was more commonly employed in period 2 (period 1, *n* = 4931, 93.9%; period 2, *n* = 1516, 98.8%; *p* < 0.001).

**Table 1:** Baseline characteristics

	Period 1 (2010–2011)		Period 2 (2014–2017)	
	Missing data		Missing data	
No. of individuals, *n*		5444		1908
BMI, mean ± SD, kg/m^2^	0	42.5 ± 5.36	0	40.2 ± 5.38
Age, mean ± SD, years	0	41.3 ± 10.96	0	40.9 ± 12.02
Comorbidity, *n* (%)	0	3257 (59.8%)	0	930 (48.7%)
Sleep apnea, *n* (%)	0	727 (13.4%)	0	208 (10.9%)
Hypertension, *n* (%)	0	1533 (28.2%)	0	416 (21.8%)
Diabetes, *n* (%)	0	959 (17.6%)	0	193 (10.1%)
Dyslipidemia, *n* (%)	0	649 (11.9%)	0	139 (7.4%)
Dyspepsia/GERD, *n* (%)	0	826 (15.2%)	0	169 (8.9%)
Depression, *n* (%)	0	819 (15.0%)	0	270 (14.2%)
Previous DVT/VTE, *n* (%)	562 (12.0%)	142 (2.6%)	0	47 (2.5%)

### Outcome

An intraoperative complication occurred in 85 (1.6%) operations during period 1 and in 25 (1.3%) operations during period 2 (*p* = 0.437).

Postoperative length of stay was on average 1.9 ± 2.57 days in period 1, and 1.6 ± 1.68 days in period 2 (*p* < 0.001).

In all, 431 (8.2%) patients suffered from any complication during period 1, and 129 (6.9%) during period 2 (OR 0.84 (0.68–1.03), *p* = 0.092; adjusted OR 0.82 (0.67–1.01), *p* = 0.068). A serious postoperative complication occurred after 192 (3.6%) operations performed during period 1 and after 46 (2.8%) operations during period 2 (OR 0.78 (0.57–1.06), *p* = 0.111; adjusted OR 0.77 (0.56–1.05), *p* = 0.098). Small bowel obstruction requiring reoperation was less common during the second period of time (67/5285, 1.3%, vs 13/1860, 0.7%, OR 0.55 (0.30–0.99), *p* = 0.045; adjusted OR 0.58 (0.32–1.06), *p* = 0.074). The risk for pulmonary complication was reduced in the second period of time (41/5285, 0.8%, vs 5/1860, 0.3%, OR 0.34 (0.14–0.87), *p* = 0.019; adjusted OR 0.33 (0–13-0.85), *p* = 0.021), other specified postoperative complications are presented in Table 2.

**Table 2:** Specified postoperative complications

	Period 1 (2010–2011)	Period 2 (2014–2017)	*p*
	Complications, *n* (%)	Complications, *n* (%)	
No. of individuals, *n*	5285	1860	
Any complication	431 (8.2%)	129 (6.9%)	0.092
Leak/intra-abdominal abscess	76 (1.4%)	14 (0.8%)	0.023
SBO/paralysis^1^	72 (1.4%)	18 (1.0%)	0.189
Bleeding	110 (2.1%)	26 (1.4%)	0.064
Other wound complication	38 (0.7%)	12 (0.6%)	0.742
Port-related complication	29 (0.5%)	2 (0.1%)	0.013
Stricture	17 (0.3%)	2 (0.1%)	0.123
Marginal ulcer	23 (0.4%)	7 (0.4%)	0.736
Cardiovascular complication	15 (0.3%)	2 (0.1%)	0.180
Pulmonary complication	41 (0.8%)	5 (0.3%)	0.019
DVT/VTE	5 (0.1%)	2 (0.1%)	1.000
Urinary tract infection	20 (0.4%)	7 (0.4%)	0.990
Other complication	83 (1.6%)	43 (2.3%)	0.037

^1^Including all cases of bowel obstruction and paralysis (grades I–V according to the Clavien-Dindo classification)

## Discussion

Closure of the mesenteric defects using non-absorbable running sutures is known to reduce the risk for internal hernia and small bowel obstruction after laparoscopic gastric bypass surgery [13, 18, 19]. When introducing this technique, there was an associated increased risk for early small bowel obstruction, mainly due to kinking of the jejunojejunostomy, and also for pulmonary complication [13]. With time, reoperation due to small bowel obstruction during the first 30 days after surgery has become less common than it was initially when introducing the mesenteric defect closure with suture technique in Sweden. The risk for pulmonary complication has also fallen. Many of the safety issues related to the suture technique may thus be attributed to a learning curve effect. However, over time, a few adaptions have been made to the technique to reduce the risk for kinking of the jejunojejunostomy. Any adaption to an established surgical procedure should preferably be assessed as part of a clinical trial [20]. Unfortunately, most adaptions have never been evaluated and scientific support for these is therefore weak [21, 22]. Bearing this in mind, some adaptions have reached wide acceptance within the Swedish surgical community and have possibly contributed to the reduction in the number of complications associated with closure of mesenteric defects with running, non-absorbable sutures.

**Fig. 1:**
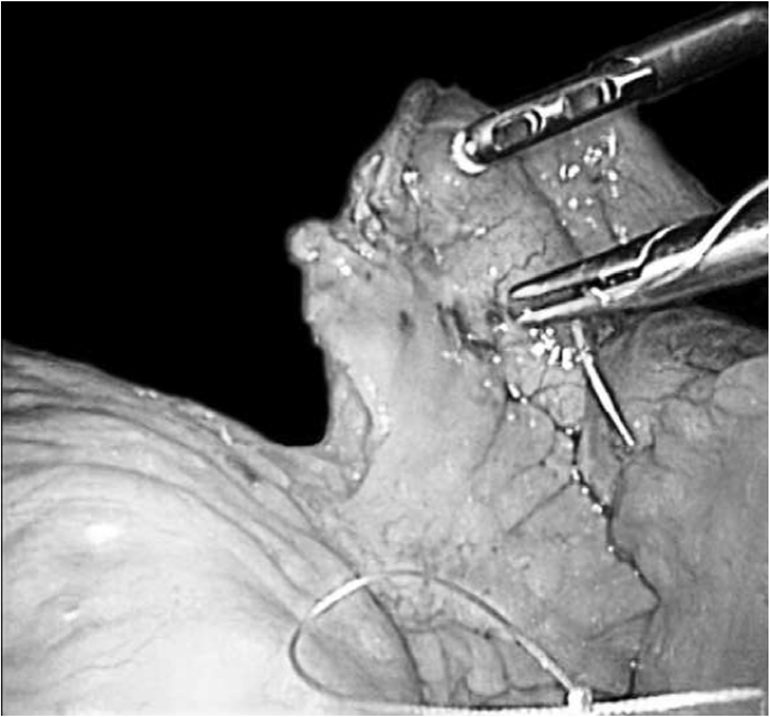
Photo illustrating closure of the mesenteric defect beneath the jejunojejunostomy

Routine division of the mesentery at the site of the blind limb next to the jejunojejunostomy (Fig. 1) is now widely accepted in Sweden [18]. The benefit of this additional step is that it creates a mobile jejunojejunostomy, located well beneath the transverse colon. This may help to reduce the strain on the anastomosis. Furthermore, with experience, many surgeons have learned to pay close attention to the sutures placed at the top of the mesenteric defect beneath the jejunojejunostomy (Fig. 1). The sutures placed close to the bowel may result in narrowing or kinking of the anastomosis, if these sutures are placed in a non-correct manner. If these sutures are placed correctly, the anastomosis will appear harmonic and the risk for kinking of the anastomosis will probably be reduced. A critical assessment of these adaptions should be made within the framework of a clinical trial. However, it is unlikely that any trial with enough power to perform such an evaluation will ever see the light of day. The present study comparing outcomes before and after introduction of mesenteric defect closure provides some support for these adaptions, and they can therefore be recommended. We also noted an increase in the application of preoperative weight loss between periods 1 and 2. This measure reduces the risk for postoperative complications after laparoscopic gastric bypass surgery [23] and is also associated with better postoperative weight loss [24]. Furthermore, it has the benefit of reducing liver size and intra-abdominal fat, thus providing better visibility during surgery [25], and significant preoperative weight loss makes it much easier to gain access to the mesenteric defects and therefore enables better and safer closure. Preoperative weight loss is now widely accepted in Sweden [15].

There are other techniques described that may help reducing the risk for kinking of the jejunojejunostomy as well. Double-stapling of the anastomosis may reduce the risk for early small bowel obstruction at the price of a slight increase in gastrointestinal bleeding [26]. An antiobstructive stitch has also been reported to have a potential preventive effect in open gastric bypass surgery [27]. The scientific support for these measures are however still weak, and although they have reached some acceptance in Sweden, they are still only used on an occasional basis.

The present study was a comparison between the outcomes of operations performed during two separate periods in time. Time generally leads to improvement in the quality and results of bariatric surgery [28] and this is perhaps the main limitation of this study. Any trial comparing surgical outcome of procedures performed during different periods in time can never exclude the impact of time itself—or rather the small unobserved improvements made over time. The main purpose of this trial, however, was to evaluate the effect of experience in mesenteric defect closure on the early complication rate after bariatric surgery. Increased experience is likely to be one of the major factors contributing to the improvement of results over time. Since we did not have information on individual surgeons, all analyses were made on a national level. The patients operated in period 2 had, on average, a lower BMI and less comorbid disease. They thus represent a slightly different group of patients than those operated within period 1. Significant early weight loss, younger age, and less comorbid disease are associated with the development of internal hernia [13]. This would imply that the healthier patients operated during period 2 were at higher risk for bowel obstruction compared to patients operated during period 1. In order to compensate for this difference, an adjusted logistic regression model was adapted. Within this model, the difference in reoperations for small bowel obstruction in the early postoperative period was no longer statistically significant. We can therefore not fully exclude that part of the difference between the two periods of time may be due to differences in patient characteristics. Finally, another limitation of this study is that many of the patients were operated outside the framework of a clinical trial and as a result, the technique for mesenteric defect closure was not standard. The surgical technique for laparoscopic gastric bypass surgery, however, is well standardized in Sweden today, and when the mesenteric defects are closed with sutures, most surgeons close the mesenteric defects with the same technique [13]. Furthermore, the effects of mesenteric defect closure in general surgical practice is also well documented [29]. Although routine closure of the mesenteric defects is well accepted throughout Sweden today, many centers have shifted their preference to metal clips instead of running, non-absorbable sutures over more recent years which explains the lower numbers during the second period. Whether or not this method is equally efficient and safe remains to be seen.

Closure of the mesenteric defects during laparoscopic gastric bypass surgery should be viewed as a routine part of the procedure to reduce the risk for internal herniation with small bowel obstruction. We have seen that once the learning curve phase has passed and adaptions are made, this technique may be safely performed.
